# Supplementary material for: Molecular Targets for Biological Therapies of Severe Asthma: Focus on Benralizumab and Tezepelumab
Source: Life (Basel). 2021 Jul 26;11(8):744. doi: 10.3390/life11080744 (PMC8399988; doi:10.3390/life11080744)
Supplement: Supplementary file 1 [file life-11-00744-s001.zip › life-1300278-supplementary.pdf]

Supplementary data

Table S1. Search strategy in PICOS.

**P**opulation	Severe Asthma
**I**ntervention	Tezepelumab of Benralizumab
**C**omparator	not specific
**O**utcome	symptom control, emergency department visits, lung function, and safety
**S**tudy design	Randomized clinical trials, observational stuides

Table S2. Search strategy in PubMed (search date：2021. 4. 19)

No.	Query	Results
1	Asthma	196,957
2	Tezepelumab	48
3	#1 AND #2	5
4	Benralizumab	411
5	#1 AND #4	27
	A total of 382 publications were identified from PubMed, while using the filters in study design*, 32 studies remains.	**32**
*Study type filter: Clinical Study, Clinical Trial, Clinical Trial, Phase I, Clinical Trial, Phase II, Clinical Trial, Phase III, Clinical Trial, Phase IV, Controlled Clinical Trial, Multicenter Study, Observational Study, Pragmatic Clinical Trial, Randomized Controlled Trial		

Table S3. Overview of Studies

List	Study name	Dates of study	Study phase	Study location or region	Interventions	Treatment arms	No. of patients randomised/ enrolled	Outcome assessment (weeks)
**Randomized controlled trials**								
1	Menzies-Gow et al. NAVIGATOR	Nov. 2017-Sep 2020	Phase 3	Asia pacific, central and eastern Europe, western Europe, Australia, North America, South America	Tezepelumab	Placebo	1061	52
						Tezepelumab		
2	Harrison et al. 2021 ANDHI	July 2017 - Sep 2019	Phase 3b	Austria, Belgium, Canada, Denmark, Finland, France, Germany, Italy, the Netherlands, Norway, Spain, Sweden, the UK, the USA	Benralizumab	Placebo	656	24
						Benralizumab 30mg q8w		
3	Emson et al. 2020 CASCADE	Sep 2018–Dec 2020	Phase 2b	Bulgaria, Czech Republic, Hungary, Israel, Japan, Latvia, Lithuania, Servia, Slovakia, South Africa, Ukraine	Tezepelumab	Low-dose Tezepelumab 70mg q4w	NA	28
						Medium-dose Tezepelumab 210mg q4w		
						High-dose Tezepelumab 280mg q2w		
						Placebo q2w		
4	Busee et al. 2018 BORA	Nov 2014-July 2016	Phase 3	Argentina, Australia, Brazil, Bulgaria, Canada, Chile, Czech Republic, France, Germany, Japan, Peru, Philippines, Poland, Romania, Russia, South Africa, South Korea, Spain, Sweden Turkey, Ukarine, the UK, US, Vietnam	Benralizumab	Benralizumab 30mg q4w	1926	56 (adults) 108 (adolescent)
						Benralizumab 30mg q8w		
5		NA	Phase 2	Bulgaria, Czech Republic, Hungary,	Tezepelumab	Low-dose Tezepelumab 70mg q4w	550	52

List	Study name	Dates of study	Study phase	Study location or region	Interventions	Treatment arms	No. of patients randomised/ enrolled	Outcome assessment (weeks)
	Corren et al. 2017 PATHWAY			Israel, Japan, Latvia, Lithuania, Servia, Slovakia, South Africa, Ukraine		Medium-dose Tezepelumab 210mg q4w		
						High-dose Tezepelumab 280mg q2w		
						Placebo q2w		
						Benralizumab		
6	Nair et al. 2017 ZONDA	April 2014–Nov 2015	Phase 2a	Argentina, Bulgaria, Canada, Chile, France, Germany, Korea, Poland, Spain, Turkey, Ukarine, US	Benralizumab	Placebo	220	28
						Benralizumab 30mg q4w		
						Benralizumab 30mg q8w		
7	Bleecker et al. 2016 SIROCCO	Sep 2013–Mar 2015	Phase 3	Africa, Asia, Europe, North America, and South America	Benralizumab	Placebo	1205	48
						Benralizumab 30mg q4w		
						Benralizumab 30mg q8w		
8	FitzGerald et al. 2016 CALIMA	August 2013–Mar 2015	Phase 3	Africa, Asia, Europe, North America, and South America	Benralizumab	Placebo	1306	56
						Benralizumab 30mg q4w		
						Benralizumab 30mg q8w		
9	Park et al. 2016	August 2011–July 2013	Phase 2a	South Korea, Japan	Benralizumab	Placebo	106	52
						Benralizumab 2mg		
						Benralizumab 20mg		
						Benralizumab 100mg		
10	Castro et al. 2014	Jan 2011-Mar2012	phase 2b	USA, Canada, Bulgaria, Brazil, Peru, Mexico, Poland, Russia, Argentina, and Colombia	Benralizumab	Eosinophilic- Placebo	324	52
						Eosinophilic- Benralizumab 2mg		
						Eosinophilic- Benralizumab 20mg		
						Eosinophilic- Benralizumab 100mg		
						Non-Eosinophilic- Placebo		
						Non-Eosinophilic- Benralizumab 100mg		
11	Nowak et al. 2015	Feb 2009–Mar 2011	Phase 2	United States, Canada	Benralizumab	Placebo	110	24 (168 days)
						Benralizumab 0.3mg/kg		
						Benralizumab 1.0mg/kg		
12	Laviolette et al. 2013	April 2008–	Phase 1	United States, Canada	Benralizumab	Placebo	27	20
						Benralizumab 100mg		

List	Study name	Dates of study	Study phase	Study location or region	Interventions	Treatment arms	No. of patients randomised/ enrolled	Outcome assessment (weeks)
		April 2011				Benralizumab 200mg		(140 days)
**Observational study**								
13	Jackson et al. 2021	2016 Nov-2020 Feb	UK Severe Asthma Registry	UK		To describe the demographic and clinical characteristics of severe asthma patients in UKSAR, to describe current biologic selection in eligible patients and to examine the difference between patients who are T2 biomarker-high and T2 biomarker-low at registration	2225	
14	Padilla-Galo et al. 2020	2019 Jan-2019 Nov.	Cross-sectional<br>multicenter	Spain	Benralizumab	To evaluate the efficacy and safety of benralizumab for the treatment of severe refractory eosinophilic asthma in a real-world cohort of patients	42	168
15	Pelaia et al. 2020		real-life<br>single center	Italy	Benralizumab	The effects of benralizumab on asthma exacerbations and lung hyperinflation	22	24
16	Menzella et al. 2019	2017 June-2019 June	Observational<br>Cross-sectional	Italy	Omalizumab, Mepolizumab Benralizumab, Bronchial themoplasty	Provide a real-life description of SRA population by describing a cohort of patients affected by refractory asthma referring to Pneumology Unit of Italy	137	

Table S4. Overview of post analysis

List	Study name	Associated references	Inclusion criteria	Treatment arm	Outcome assessment
**Post analysis**					
17	Corren et al. 2021	PATHWAY Phase 2b	The same as PATHWAY	Low-dose Tezepelumab 70mg q4w<br>Medium-dose Tezepelumab 210mg q4w<br>High-dose Tezepelumab 280mg q2w<br>Placebo q2w	Further assessed the impact of tezepelumab on patient-reported outcomes in PATHWAY
18	Corren et al. 2020	PATHWAY Phase 2b	The same as PATHWAY	Low-dose Tezepelumab 70mg q4w<br>Medium-dose Tezepelumab 210mg q4w<br>High-dose Tezepelumab 280mg q2w<br>Placebo q2w	evaluated tezepelumab's effect on the frequency and duration of hospitalization, ED visits, and ICU visits
19	Lugogo et al. 2020	SIROCCO CALIMA	aged ≥ 18 years	Benralizumab 30mg q4w<br>Benralizumab 30mg q8w<br>Placebo q4w	blood eosinophil count (BECs) were measured at baseline and weeks 4, 8, 24, 40, and at end of treatment week 48 for SIROCCO, and week 56 for CALIMA
20	Jackson et al. 2020	SIROCCO CALIMA	The same as SIROCCO and CALIMA	Benralizumab 30mg q4w<br>Benralizumab 30mg q8w<br>Placebo q4w	evaluated the predicted value of baseline blood eosinophil counts vs. serum IgE concentrations on exacerbation risk and the association of these variables with benralizumab treatment effect
21	Chipps et al. 2020	SIROCCO CALIMA	Patients who were receiving high-dose ICS/LABA, representing patients receiving Global Initiative for Asthma Steps 4 and 5 treatment, and had baseline eosinophil counts of at least 300 cells/uL	Benralizumab 30mg q4w<br>Benralizumab 30mg q8w<br>Placebo q4w	Evaluated fixed airflow obstruction influence on benralizumab treatment response
22	Chia et al. 2019	SIROCCO CALIMA	The same as SIROCCO and CALIMA	Benralizumab 30mg q4w<br>Benralizumab 30mg q8w<br>Placebo q4w	PK exposure and efficacy endpoints of AER
23	Chupp et al. 2019	SIROCCO CALIMA ZONDA	The same as SIROCCO and CALIMA and ZONDA	Benralizumab 30mg q4w<br>Benralizumab 30mg q8w<br>Placebo q4w	To assess benralizumab's onset of action and efficacy by examining change in morning peak expiratory flow (PEF) after initiation of treatment in the phase 3 SIROCCO, CALIMA, and Zonda

List	Study name	Associated references	Inclusion criteria	Treatment arm	Outcome assessment
24	Bleecker et al. 2018	SIROCCO CALIMA	High-dosage ICS/LABA	Benralizumab 30mg q4w	Baseline clinical factors and blood eosinophil counts can help identify patients potentially responsive to benralizumab
				Benralizumab 30mg q8w	
				Placebo q4w	
25	DuBuske et al. 2018	SIROCCO CALIMA	The same as SIROCCO and CALIMA	Benralizumab 30mg q4w	Evaluated the effect of benralizumab treatment on seasonal asthma exacerbation rates
				Benralizumab 30mg q8w	
				Placebo q4w	
26	Chipps et al. 2018	SIROCCO CALIMA	Africa, Asia, Europe, North America, and South America	Benralizumab 30mg q4w	To investigate the efficacy of benralizumab by atopic status and serum IgE concentrations
				Benralizumab 30mg q8w	
				Placebo q4w	
27	Sehmi et al. 2018	ZONDA Phase 3	From 4 academic centers, we recruited 18 patients with severe asthma and elevated blood eosinophils who relied on OCS therapy to control their disease	Benralizumab 30mg q4w	28
				Benralizumab 30mg q8w	
				Placebo q4w	
28	Ohta et al. 2018	CALIMA	Analysis of the subset of Japanese patients who participated in the CALIMA trial	Benralizumab 30mg q4w	56
				Benralizumab 30mg q8w	
				Placebo q4w	
29	Goldman et al. 2017	SIROCCO CALIMA	aged ≥ 18 years	Placebo	
				Benralizumab 30mg q8w	
30	Pham et al. 2016	Phase I (MI-CP166) Phase IIa (MI-CP197)	Eligible patients aged 20-68 years with asthma were enrolled in two independent, randomized, double-blind, placebo-controlled clinical trials	Placebo	52
				Benralizumab 25mg	
				Benralizumab 100mg	
				Benralizumab 200mg	

Table S5. Baseline Characteristics

List	Trial	Treatment Arm		N	Age, mean, years (SD)	Race, (n)	Gender, n	BMI, mean, (SD)	FEV_1 on dosing date		FEV_1/FVC	ACQ-6 score (SD)	AQLQ +12 score (SD)
									liters	% predicted			
**Randomized controlled trials**													
1	Menzies-Gow et al. 2021	Tezepelumab		528	49.9 (16.3)	W:332, O:196	M:193 F: 335	28.7 (7.1)	1.8 (0.7)	62.8±18.0	-	2.8 (0.8)	3.9 (1.0)
		Placebo		531	49.0 (15.9)	W:327; O:204	M:531 F:337	28.3 (6.9)	1.9 (0.7)	62.7±18.0	-	2.8 (0.8)	3.9 (1.0)
2	Harrison et al. 2020	Benralizuamb		427	52.5 (12.7)	W:314; B:35; A:11; O:8; H:49; M:62	M:164 F:263	29.85 (7.37)	1.63 (0.609)	54.0±14.2	-	3.04 (0.874)	-
		Placebo		229	53.3 (12.5)	W:168; B:18; A:7 O:2; H:25; M:33	M:93 F:136	30.10 (7.89)	1.72 (0.629)	55.9±13.6	-	3.07 (0.965)	-
3	Emson et al. 2020	Nasal polyposis+	Tezepelumab	23	51.3 (12.7)	NA	M:8 F:15	NA	1.95 (0.73)	-	-	2.85 (0.91)	-
			Placebo	18	55.9 (9.2)	NA	M:8 F:10	NA	2.00 (0.60)	-	-	2.51 (0.91)	-
		Nasal polyposis-	Tezepelumab	112	51.3 (12.7)	NA	M:42 F:70	NA	1.81 (0.55)	-	-	2.69 (0.78)	-
			Placebo	117	53.1 (12.6)	NA	M:35 F:82	NA	1.80 (0.59)	-	-	2.67 (0.65)	-
4	Busse et al., 2019	Benralizuamb 30mg Q4W	Q4W/Q4W	518	51.3 (13.3)	W:398; B:6 A:94; O:20	M:179 F:339	29.0 (7.0)	1.95 (0.70)	67.1±17.9	63.5±11.7	1.5 (1.2)	5.39 (1.19)
			Plabo/Q4W	265	49.9 (14.7)	W:201; B:10 A:44; O:10	M:101 F:164	29.1 (6.7)	1.89 (0.79)	62.8±19.7	62.0±13.7	1.6 (1.2)	5.28 (1.32)

List	Trial	Treatment Arm		N	Age, mean, years (SD)	Race, (n)	Gender, n	BMI, mean, (SD)	FEV$_1$ on dosing date liters	FEV$_1$ on dosing date % predicted	FEV$_1$/FVC	ACQ-6 score (SD)	AQLQ +12 score (SD)
			Total	783	51.1 (13.8)	W:599; B:16 A:138; O:30	M:280 F:503	29.1 (6.9)	1.93 (0.73)	65.7±18.6	63.0±12.4	1.5 (1.2)	5.36 (1.23)
		Benralizuamb 30mg Q8W	Q8W/Q8W	512	49.3 (14.8)	W:388; B:12 A:92; O:20	M:205 F:307	28.4 (6.2)	2.02 (0.75)	66.9±19.0	63.0±13.3	1.4 (1.1)	5.48 (1.14)
			Plabo/Q8W	281	48.2 (14.8)	W:224; B:7 A:44; O:6	M:118 F:163	28.9 (7.1)	2.00 (0.82)	66.2±20.0	64.0±13.2	1.6 (1.2)	5.27 (1.21)
			Total	793	48.9 (15.5)	W:612; B:19 A:136; O:26	M:323 F:470	28.6 (6.5)	2.01 (0.78)	66.7±19.3	63.0±13.3	1.5 (1.1)	5.40 (1.17)
5	Corren 2017	Placebo		138	52.3 (11.7)	W:123; B:6 A:6; O:2	M: 44 F: 94	28.5 (5.6)	1.82 (0.59)	60.0±13.5	NA	2.66 (0.69)	4.09 (0.87)
		Low-dose Tezepelumab		138	50.8 (12.4)	W:131; B:4 A:3; O:0	M: 49 F: 89	28.3 (5.1)	1.91 (0.67)	60.0±13.7	NA	2.72 (0.79)	4.17 (0.93)
		Medium-Dose Tezepelumab		137	52.7 (12.3)	W:128; B:3 A:5; O:0	M: 50 F: 87	28.5 (4.9)	1.83 (0.58)	59.0±12.5	NA	2.70 (0.80)	4.20 (0.91)
		High-dose Tezepelumab		137	51.3 (12.4)	W:381; B:13 A:13; O:2	M: 46 F: 91	27.6 (5.0)	1.83 (0.57)	58.8±11.8	NA	2.64 (0.74)	4.08 (0.91)
			Benralizumab	28	46.4 (16.0)	W:28; B:0 A:0; O:0	M:14 F:14	28.6 (7.3)	2.403 (0.71)	70.0±12.8	67±9	NA	NA
		Baseline blood eosinophil	Placebo	72	51.8 (12.3)	W:68; B:2 A:0; O:2	M:26 F:46	29.5 (6.9)	2.247 (0.805)	71.7±12.8	67±10	NA	NA

List	Trial	Treatment Arm		N	Age, mean, years (SD)	Race, (n)	Gender, n	BMI, mean, (SD)	FEV$_1$ on dosing date		FEV$_1$/FVC	ACQ-6 score (SD)	AQLQ +12 score (SD)
									liters	% predicted			
		count < 300 cells per uL	Benralizumab	78	49.0 (13.8)	W:70; B:7 A:1; O:0	M:30 F:48	30.2 (7.0)	2.193 (0.559)	69.5±10.8	69±9	NA	NA
6	Nair 2017	Placebo		75	49.9 (11.7)	NA	M: 27 F: 48	28.7 (5.2)	1.93 (0.662)	62.0±16.5	62±13	2.7 (1)	4.1 (1.1)
		Benralizumab q4w		72	50.2 (12.0)	NA	M: 32 F: 40	29.8 (6.8)	1.85 (0.741)	57.4±18	59±13	2.6 (1.1)	4.2 (1.1)
		Benralizumab q8w		73	52.9 (10.1)	NA	M: 26 F: 47	30.2 (6.5)	1.75 (0.635)	59.0±17.9	59±12	2.4 (1.2)	4.4 (1.2)
7	Bleecker 2016	High-dosage ICS/LABA with baseline blood eosinophils ≥ 300 cells per uL	Placebo	267	48.6 (14.7)	W:191; B:10 A:36; O:30	M: 87 F: 180	28.7 (7.0)	1.654	56.4	61±13	2.90 (0.95)	3.87 (0.99)
			Benralizumab 30mg q4w	275	49.2 (13.1)	W:191; B:11 A:39; O:34	M: 102 F: 173	28.9 (6.9)	1.673	56.5	62±12	2.77 (0.95)	3.93 (1.00)
			Benralizumab 30mg q8w	267	47.6 (14.6)	W:192; B:10 A:35; O:30	M: 93 F: 174	27.7 (6.1)	1.66	55.5	60±13	2.81 (0.89)	3.93 (0.97)
		High-dosage ICS/LABA with baseline blood eosinophils < 300 cells per uL	Placebo	140	49.0 (15.3)	W:111; B:6 A:14; O:9	M: 51 F: 89	29.3 (7.1)	1.672	57	62±13	2.82 (0.93)	3.97 (1.07)
			Benralizumab 30mg q4w	124	52.0 (13.9)	W:94; B:4 A:15; O:11	M: 22 F: 102	29.9 (7.3)	1.615	59.4	63±12	2.78 (1.00)	3.92 (0.95)
			Benralizumab 30mg q8w	131	47.8 (14.3)	W:95; B:5 A:15; O:16	M: 53 F: 78	29.3 (6.2)	1.721	57.3	62±14	2.78 (0.85)	3.97 (1.04)
8	FitzGerald 2016	High-dosage ICS/LABA	Placebo	248	48.5 (14.1)	W:213; B:8	M: 103 F: 145	29.0 (6.1)[1]	1.815	58.2	60±12	2.75 (0.94)	3.93 (1.04)

[1] Data not available for all randomized patients

List	Trial	Treatment Arm		N	Age, mean, years (SD)	Race, (n)	Gender, n	BMI, mean, (SD)	FEV_1 on dosing date liters	% predicted	FEV_1/FVC	ACQ-6 score (SD)	AQLQ +12 score (SD)
		with baseline blood eosinophils ≥ 300 cells per uL				A:27; O:0							
			Benralizumab 30mg q4w	241	50.1 (13.1)	W:209; B:5 A:27; O:0	M: 82 F: 159	29.1 (7.3)[1]	1.75	59.1	61±12	2.70 (0.91)	3.99 (0.98)
			Benralizumab 30mg q8w	239	49.6 (13.0)	W:203; B:8 A:28; O:0	M: 101 F: 138	28.6 (6.1)[1]	1.758	57	60±13	2.80 (0.95)	3.87 (1.05)
		High-dosage ICS plus LABA with baseline blood eosinophils < 300 cells per uL	Placebo	122	52.4 (14.4)	W:108; B:4 A:10; O:0	M: 46 F: 76	29.7 (7.4)[1]	1.639	56.1	60±13	2.68 (0.89)	4.03 (1.01)
			Benralizumab 30mg q4w	116	51.9 (12.2)	W:99; B:3 A:14; O:0	M: 45 F: 71	28.8 (6.5)[1]	1.717	57.4	59±13	2.82 (0.89)	3.95 (0.93)
			Benralizumab 30mg q8w	125	51.1 (13.8)	W:107; B:5 A:12; O:1	M: 38 F: 87	29.8 (7.2)[1]	1.665	56.7	60±14	2.87 (0.96)	3.82 (0.97)
9	Park 2016	Placebo		26	50.8 (11.8)	J:11 S:15	M: 9 F: 17	24.4	NA	68.8±16.3[2]	65.2±13.7[3]	1.6 (0.6)	NA
		Benralizumab 2mg		26	53.2 (11.3)	J:11 S:15	M: 13 F: 13	24.9	NA	65.3±14.1[2]	66.3±12[3]	1.8 (0.6)	NA
		Benralizumab 20mg		25	55.6 (8.9)	J:11 S:14	M: 6 F: 19	25.3	NA	70.6±13.2[2]	68.6±10.8[3]	1.9 (0.7)	NA
		Benralizumab 100mg		26	51.4 (13.8)	J:11 S:15	M: 10 F: 16	24.5	NA	67.5±15.8[2]	67.8±9.3[3]	1.9 (0.8)	NA
10	Castro et al. 2014	Eosinophilic[4]	Placebo	80	45.6 (11.7)	W: 66%	M:27 F:53	28.8 (6.0)	NA	65.0±15.3	62.1±11.3	2.7 (1.0)	3.6 (1.2)

[2] N=24 in each group, FEV_1 (actual)/ FEV_1 (predicted)*100, data that did not meet ATS/ERS criteria were deleted
[3] N=24 in each group, data that did not meet ATS/ERS criteria were deleted, FEV_1/ FVC *100,
[4] Eosinophilic refers to ELEN index positive or FeNO≥50 ppb; non-eosinophilic refers to ELEN index negative and FeNO<50 ppb

List	Trial	Treatment Arm		N	Age, mean, years (SD)	Race, (n)	Gender, n	BMI, mean, (SD)	FEV_1 on dosing date liters	FEV_1 on dosing date % predicted	FEV_1/FVC	ACQ-6 score (SD)	AQLQ +12 score (SD)
			Benralizumab 2mg	81	47.1 (12.8)	W: 73%	M:23 F:58	29.2 (6.5)	NA	65.1±15.2	64.9±11.0	2.6 (1.0)	3.7 (1.2)
			Benralizumab 20mg	81	46.6 (13.2)	W: 62%	M:33 F:48	28.0 (5.2)	NA	64.8±14.8	63.3±10.9	2.5 (0.9)	3.8 (1.1)
			Benralizumab 100mg	82	47.8 (12.9)	W: 76%	M:22 F:60	29.6 (5.0)	NA	66.1±15.9	63.9±11.5	2.5 (1.0)	3.7 (1.0)
		Non-eosinophilic	Placebo	142	50.0 (12.3)	W: 75%	M:42 F:100	29.5 (6.0)	NA	69.1±14.5	66.9±11.4	2.5 (0.8)	3.8 (1.0)
			Benralizumab 100mg	140	50.0 (11.5)	W: 71%	M:42 F:98	30.2 (7.0)	NA	66.8±15.1	65.1±11.4	2.6 (0.8)	3.7 (1.0)
11	Nowak 2015	Placebo		38	Mean (Range): 35.9 (22-55)	W:11; B:26 A:0; O:1	M: 8 F: 30	34.7 (8.9)	1.68	56.1	NA	3.64 (0.83)	3.10 (0.90)
		Benralizumab 0.3mg/kg		36	Mean (Range): 37.9 (20-60)	W:14; B:19 A:0; O:3	M: 12 F: 24	30.1 (8.1)	1.69	53.1	NA	3.72 (1.18)	3.18 (0.95)
		Benralizumab 1.0mg/kg		36	Mean (Range): 34.8 (19-54)	W:16; B:19 A:0; O:1	M: 13 F: 23	30.8 (8.3)	2.07	63.3	NA	3.26 (1.27)	3.35 (1.11)
		Benralizumab Total		72	Mean (Range): 36.3 (19-60)	W:30; B:38 A:0; O:4	M: 25 F: 47	30.5‡ (8.2)	1.88‡	58.1	NA	3.49 (1.24)	3.27 (1.03)
12	Laviolette 2013	Cohort 1 (single-dose IV)	Placebo	5	34.4 (9.7)	W:4 O:1	M: 3 F: 2	NA	2.5 (0.8)	67.2±9.1[5]	66.7±10	NA	NA
			Benralizumab 1mg/kg	8	38.9 (14.7)	W:8 O:0	M: 2 F: 6	NA	2.3 (0.6)	70.5±15.6	70.7±9.8	NA	NA
		Cohort 2 (multiple-dose SC)	Placebo	5	40.2 (14.5)	W:5 O:0	M: 1 F: 4	NA	1.9 (0.4)	60.5±6.6	67±12.7	NA	NA
			Benralizumab 100mg	4	41.0 (14.9)	W:4 O:0	M: 3 F: 1	NA	3.0 (1.2)	72.5±13.1	69.6±5.9	NA	NA
			Benralizumab 200mg	5	37.2 (14.4)	W:4 O:1	M: 2 F: 3	NA	2.2 (0.6)	65.7±10.2	67.2±4.2	NA	NA
			Benralizumab Total	9	38.9 (13.8)	W:8 O:1	M: 5 F: 4	NA	2.6 (1.0)	68.7±11.4	68.3±4.8	NA	NA

[5] Screening postbronchodilator FEV_1 predicted (%) and screening prebronchodilator FEV_1/FVC(%) were used as part of the eligibility criteria

List	Trial	Treatment Arm	N	Age, mean, years (SD)	Race, (n)	Gender, n	BMI, mean, (SD)	FEV1 on dosing date liters	FEV1 on dosing date % predicted	FEV1/FVC	ACQ-6 score (SD)	AQLQ +12 score (SD)
**Post-analysis**												
19	Lugogo et al. 2020	BEC maintenance	260	51.2 (12.9)	W:209; B:9 A:34; O:8	M:85 F:175	28.6 (6.3)	1.639±0.544	55.8±13.9	59.5±12.4	2.8 (0.9)	NA
		BEC group shift	474	50.7 (12.7)	W:378; B:18 A:52; O:26	M:174 F:300	29.8 (6.9)	1.687±0.610	56.3±14.6	60.5±12.5	2.8 (0.9)	NA
21	Chipps et al. 2020	FAO+	935	51.3 (12.5)	W:745; B:29 A:114; O:47	M:384 F:551	28.6 (6.1)	1.569±0.569	52.0±13.6	NA	2.8 (0.9)	3.9 (1.0)
		FAO-	558	44.7 (14.9)	W:416; B:19 A:77; O:46	M:175 F:383	28.7 (7.2)	1.961±0.568	65.2±11.3	NA	2.7 (1.0)	4.0 (1.0)
24	Bleecker et al. 2018	Placebo	777	49.2 (14.6)	W:623; B:28 A:87; O:39	M:287 F:490	29.1 (6.8)	1.71±0.61	57.0±14.9	61±13	2.8 (0.9)	3.9 (1.0)
		Benralizumab 30mg q4w	756	50.4 (13.2)	W:593; B:23 A:95; O:45	M:251 F:505	29.1 (7.0)	1.70±0.57	57.9±14.3	61±12	2.8 (0.9)	4.0 (1.0)
		Benralizumab 30mg q8w	762	48.8 (14.0)	W:597; B:28 A:90; O:47	M:285 F:477	28.6 (6.3)	1.70±0.60	56.5±14.6	60±13	2.8 (0.9)	3.9 (1.0)
26	Chipps et al. 2018	Met atopy and IgE 30-700kU/L criteria	824	47.3 (14.2)	W:611; B:34 A:122; O:57	M:308 F:516	28.5 (6.4)	1.754±0.607	57.9±14.2	NA	2.7 (0.9)	4.0 (1.0)
		Did not meet atopy and IgE 30-700kU/L criteria	1471	50.7 (13.6)	W:1202; B:45 A:150; O:74	M:515 F:936	29.2 (6.9)	1.671±0.587	56.7±14.8	NA	2.8 (0.9)	3.9 (1.0)

List	Trial	Treatment Arm		N	Age, mean, years (SD)	Race, (n)	Gender, n	BMI, mean, (SD)	FEV_1 on dosing date liters	% predicted	FEV_1/FVC	ACQ-6 score (SD)	AQLQ +12 score (SD)
		IgE high (≥150kU/L)		1204	47.7 (14.5)	W:887; B:52 A:176; O:89	M:488 F:716	28.7 (6.8)	1.748±0.614	57.5±14.6	NA	2.8 (0.9)	4.0 (1.0)
		IgE low		1053	51.4 (13.1)	W:896; B:26 A:92; O:39	M:316 F:737	29.3 (6.8)	1.650±0.568	56.9±14.5	NA	2.8 (0.9)	3.9 (1.0)
		Atopy		1375	46.8 (14.6)	W:1018; B:63 A:196; O:98	M:521 F:824	29.0 (7.0)	1.775±0.615	58.0±14.4	NA	2.7 (0.9)	4.0 (1.0)
		No atopy		883	53.5 (12.0)	W:768; B:15 A:72; O:28	M:256 F:627	28.9 (6.3)	1.588±0.541	56.0±14.7	NA	2.9 (0.9)	3.8 (1.0)
28	Ohta 2018	Placebo		16	Median(Range): 47 (29-70)	A:16	M:3 F:13	27.5 (4.8)	1.616±0.652	61.9±11.4	60±10	NA	NA
		Benralizumab q4w		15	Median(Range): 54(34-72)	A:15	M:5 F:10	24.5 (4.2)	1.466±0.488	57.7±15.6	63±9	NA	NA
		Benralizumab q8w		15	Median(Range): 49 (31-66)	A:15	M:6 F:9	25.3 (3.9)	1.721±0.506	61.3±12.5	66±12	NA	NA
29	Goldman et al. 2017	[SIROCCO] High-dosage ICS/LABA with baseline blood eosinophils ≥ 150 cells per uL	Placebo	306	50.8 (12.6)	W:221; B:12 A:41; O:32	M:96 F:210	29.0 (6.8)	1.600±0.553	55.1±14.3	61±13	2.90 (0.96)	3.89 (1.01)
			Benralizumab 30mg Q8W	325	49.6 (12.7)	W:239; B:11 A:40; O:35	M:114 F:211	28.4 (5.9)	1.665±0.576	55.6±14.3	60±12	2.82 (0.88)	3.90 (0.97)
		[SIROCCO] High-dosage ICS/LABA with baseline blood eosinophils <	Placebo	74	51.6 (12.0)	W:59; B:3 A:8; O:4	M:25 F:49	30.8 (7.5)	1.664±0.606	56.9±15.4	60±12	2.85 (0.90)	3.79 (1.01)
			Benralizumab 30mg Q8W	48	48.3 (12.6)	W:30; B:2 A:9; O:7	M:19 F:29	29.2 (5.2)	1.590±0.518	54.3±15.1	62±14	2.72 (0.88)	4.01 (1.11)

List	Trial	Treatment Arm		N	Age, mean, years (SD)	Race, (n)	Gender, n	BMI, mean, (SD)	FEV$_1$ on dosing date liters	FEV$_1$ on dosing date % predicted	FEV$_1$/FVC	ACQ-6 score (SD)	AQLQ +12 score (SD)
		150 cells per uL											
		[CALIMA] High-dosage ICS/LABA with baseline blood eosinophils ≥ 150 cells per uL	Placebo	315	50.6 (12.9)	W:273; B:11 A:31; O:0	M:125 F:190	29.6 (6.4)	1.759±0.614	57.7±14.1	60±12	2.71 (0.92)	3.96 (1.03)
			Benralizumab 30mg Q8W	300	51.0 (11.8)	W:255; B:12 A:32; O:1	M:119 F:181	29.1 (6.1)	1.720±0.623	56.3±14.3	59±13	2.81 (0.93)	3.86 (1.02)
		[CALIMA] High-dosage ICS plus LABA with baseline blood eosinophils < 150 cells per uL	Placebo	40	53.3 (13.5)	W:34; B:0 A:6; O:0	M:15 F:25	28.5 (7.6)	1.474±0.535	50.4±15.5	57±14	2.92 (0.86)	3.89 (0.89)
			Benralizumab 30mg Q8W	48	52.3 (13.5)	W:42; B:0 A:6; O:0	M:14 F:34	29.1 (7.5)	1.710±0.614	58.6±14.1	61±16	2.89 (1.05)	3.79 (0.95)
30	Pham 2016	MI-CP166	Placebo	5	Median (Range): 37.0 (23-60)	W:5	M:1 F:4	Median (Range): 25.5 (21.7-40.9)	Median (Range): 1.8 (1.36-4.6)	60.5±6.6	Median (Range): 70.2 (45.0-77.0)	NA	NA
			Benralizumab 100mg	4	Median (Range): 45.5 (20-53)	W:4	M:3 F:1	Median (Range): 29.3 (25.0-34.5)	Median (Range): 2.9 (1.7-4.6)	72.5±13.1	Median (Range): 71.0 (60.0-74.1)	NA	NA
			Benralizumab 200mg	5	Median (Range): 32.0 (26-62)	W:4 O:1	M:2 F:3	Median (Range): 31.2 (22.7-32.0)	Median (Range): 2.5 (1.4-2.9)	65.7±10.2	Median (Range): 69.3 (60.2-71.4)	NA	NA

List	Trial	Treatment Arm		N	Age, mean, years (SD)	Race, (n)	Gender, n	BMI, mean, (SD)	FEV_1 on dosing date liters	% predicted	FEV_1/FVC	ACQ-6 score (SD)	AQLQ +12 score (SD)
		MI-CP197	Placebo	6	Median (Range): 44.0 (30-60)	W:4 O:2	M: 3 F: 3	Median (Range): 27.8 (25.0-36.1)	Median (Range): 3.0 (2.0-4.1)	Median (Range): 88.0 (58.0-111.0)	Median (Range): 70.0 (62.0-79.0)	NA	NA
			Benralizumab 25mg	6	Median (Range): 49.0 (36-64)	W:5 O:1	M: 3 F: 3	Median (Range): 30.0 (23.4-38.8)	Median (Range): 2.6 (1.7-3.2)	Median (Range): 75.0 (61.0-81.0)	Median (Range): 68.0 (56.0-79.0)	NA	NA
			Benralizumab 100mg	6	Median (Range): 44.0 (31-68)	W:4 O:2	M: 2 F: 4	Median (Range): 25.8 (20.9-44.5)	Median (Range): 2.4 (2.2-2.8)	Median (Range): 82.0 (61.0-87.0)	Median (Range): 73.0 (61.0-84.0)	NA	NA
			Benralizumab 200mg	6	Median (Range): 35.0 (27-62)	W:6 O:0	M: 3 F: 3	Median (Range): 28.9 (25.0-32.9)	Median (Range): 2.4 (2.0-2.8)	Median (Range): 67.0 (61.0-76.0)	Median (Range): 66.0 (62.0-72.0)	NA	NA

Table S6 Summary of Annual Exacerbation rate

List	Trial	Treatment arm		Subjects	Crude annual rate (95% CI)	Absolute difference estimate	Relative reduction vs. placebo	p value vs. placebo
1	Menzies-Gow et al. 2021	≥ 300 Eosinophils per uL	Placebo	222	2.66		-	
			Tezepelumab	219	0.79		0.30 (0.22-0.40)	
		< 300 Eosinophils per uL	Placebo	309	1.73		-	
			Tezepelumab	309	1.02		0.59 (0.46-0.75)	
		≥ 150 Eosinophils per uL	Placebo	393	2.24		-	
			Tezepelumab	390	0.89		0.39 (0.32-0.49)	
			Placebo	138	1.70		-	

List	Trial	Treatment arm		Subjects	Crude annual rate (95% CI)	Absolute difference estimate	Relative reduction vs. placebo	p value vs. placebo
		< 150 Eosinophils per uL	Tezepelumab	138	1.04		0.61 (0.42-0.88)	
		FeNO ≥ 25	Placebo	307	2.52		-	
			Tezepelumab	309	0.82		0.32 (0.25-0.42)	
		FeNO < 25	Placebo	220	1.57		-	
			Tezepelumab	213	1.07		0.68 (0.51-0.92)	
3	Emson et al. 2020	NP+	Placebo	18	1.08		-	
			Tezepelumab	23	0.29		0.25 (0.07-0.85)	
		NP-	Placebo	117	0.68		-	
			Tezepelumab	112	0.19		0.27 (0.14-0.53)	
4	Busse et al. 2018	≥ 300 cells per uL	Q4W/Q4W group	347	0.48 (0.42-0.56)			
			Placebo/Q4W group	172	0.53 (0.43-0.65)			
			Total	519	0.50 (0.44-0.56)			
			Q8W/Q8W group	339	0.46 (0.39-0.53)			
			Placebo/Q8W group	188	0.57 (0.47-0.68)			
			Total	527	0.49 (0.44-0.56)			
		< 300 cells per uL	Q4W/Q4W group	171	0.74 (0.62-0.88)			
			Placebo/Q4W group	93	0.80 (0.64-1.00)			
			Total	264	0.76 (0.66-0.87)			
			Q8W/Q8W group	173	0.59 (0.49-0.71)			
			Placebo/Q8W group	93	0.74 (0.59-0.94)			
			Total	266	0.64 (0.55-0.74)			
5		Total	Placebo	138	0.72 [0.61 – 0.86][6]		-	-

[6] 90% CI

List	Trial	Treatment arm		Subjects	Crude annual rate (95% CI)	Absolute difference estimate	Relative reduction vs. placebo	p value vs. placebo
	Corren et al. 2017		Tezepelumab 70mg q4w	138	0.27 [0.20 – 0.36][6]		0.62 (0.42-0.75)[6]	<0.001
			Tezepelumab 210mg q4w	137	0.20 [0.14 – 0.28][6]		0.71 (0.54-0.82)[6]	<0.001
			Tezepelumab 280mg q2w	137	0.23 [0.17 – 0.32][6]		0.66 (0.47-0.79)[6]	<0.001
		≥ 250 Eosinophils per uL	Placebo	78	0.78 [0.59 – 1.00]		-	-
			Tezepelumab 70mg q4w	80	0.29 [0.18 – 0.43]		0.65 (0.30-0.82)	0.003
			Tezepelumab 210mg q4w	76	0.26 [0.16 – 0.42]		0.65 (0.27-0.83)	0.005
			Tezepelumab 280mg q2w	76	0.21 [0.12 – 0.35]		0.72 (0.40-0.87)	0.001
		< 250 Eosinophils per uL	Placebo	60	0.65 [0.46 – 0.89]		-	-
			Tezepelumab 70mg q4w	58	0.25 [0.14 – 0.42]		0.60 (0.12-0.81)	0.022
			Tezepelumab 210mg q4w	61	0.14 [0.06 – 0.27]		0.79 (0.48-0.92)	<0.001
			Tezepelumab 280mg q2w	61	0.26 [0.14 – 0.43]		0.58 (0.11-0.80)	0.024
7	Bleecker et al. 2016	High-dosage ICS /LABA with baseline blood	Placebo	267	1.33 (1.12-1.58)[7]	-	-	-
			Benralizumab 30mg Q4W	275	0.73 (0.60-0.89)[7]	-0.60 (-0.87 to -0.33)	0.55 (0.42 to 0.71)[8]	<0.0001

[7] Estimates calculated using a negative binomial model, with adjustment for treatment, region, oral corticosteroid use at time of randomization, and previous exacerbations
[8] Rate ratio vs. placebo

List	Trial	Treatment arm		Subjects	Crude annual rate (95% CI)	Absolute difference estimate	Relative reduction vs. placebo	p value vs. placebo
		eosinophils ≥ 300 cells per uL	Benralizumab 30mg Q8W	267	0.65 (0.53-0.80)[7]	-0.68 (-0.95 to -0.42)	0.49 (0.37 to 0.64)[8]	<0.0001
		High-dosage ICS/ LABA with baseline blood eosinophils < 300 cells per uL	Placebo	140	1.21 (0.96-1.52)[7]	-	-	-
			Benralizumab 30mg Q4W	124	0.85 (0.65-1.11)[7]	-0.36 (-0.71 to 0.00)	0.70 (0.50 to 1.00)[8]	0.0471
			Benralizumab 30mg Q8W	131	1.00 (0.78-1.28)[7]	-0.21 (-0.58 to 0.16)	0.83 (0.59 to 1.16)[8]	0.2685
8	FitzGerald et al. 2016	High-dosage ICS plus LABA with baseline blood eosinophils ≥ 300 cells per uL	Placebo	248	0.93 (0.77-1.12)[7]	-	-	-
			Benralizumab 30mg Q4W	241	0.60 (0.48-0.74)[7]	-0.33 (-0.54 to -0.12)	0.64 (0.49 to 0.85)	0.0018
			Benralizumab 30mg Q8W	239	0.66 (0.54-0.82)[7]	-0.26 (-0.48 to -0.04)	0.72 (0.54 to 0.95)	0.0188
		High-dosage ICS plus LABA with baseline blood eosinophils < 300 cells per uL	Placebo	122	1.21 (0.96-1.52)[7]	-	-	-
			Benralizumab 30mg Q4W	116	0.78 (0.59-1.02)[7]	-0.43 (-0.78 to -0.08)	0.64 (0.45 to 0.92)	0.015
			Benralizumab 30mg Q8W	125	0.73 (0.55-0.95)[7]	-0.48 (-0.82 to -0.14)	0.60 (0.42 to 0.86)	0.0048
10	Castro et al. 2014	Eosinophilic	Placebo	80	Mean (SD) 0.57 (0.75)		-	-
			Benralizumab 2mg	81	Mean (SD) 0.65 (0.81)		-0.09 (-0.59-0.26)[9]	0.781
			Benralizumab 20mg	81	Mean (SD) 0.37 (0.61)		0.36 (0.03-0.58)[9]	0.173
			Benralizumab 100mg	82	Mean (SD) 0.34 (0.58)		0.41 (0.11-0.60)[9]	0.096
		Non-eosinophilic	Placebo	142	Mean (SD) 0.56 (0.75)		-	-
			Benralizumab 100mg	140	Mean (SD) 0.43 (0.66)		0.22 (-0.05-0.42)[9]	0.284

[9] 80% CI

List	Trial	Treatment arm		Subjects	Crude annual rate (95% CI)	Absolute difference estimate	Relative reduction vs. placebo	p value vs. placebo
		Baseline blood eosinophil count ≥300 cells per uL	Placebo	83	Mean (SD) 0.68 (0.82)		-	-
			Benralizumab 2mg	65	Mean (SD) 0.75 (0.87)		-0.07 (-0.55-0.26)[9]	0.822
			Benralizumab 20mg	70	Mean (SD) 0.30 (0.55)		0.57 (0.33-0.72)[9]	0.015
			Benralizumab 100mg	97	Mean (SD) 0.38 (0.62)		0.43 (0.18-0.60)[9]	0.049
		Baseline blood eosinophil count <300 cells per uL	Placebo	139	Mean (SD) 0.49 (0.70)		-	-
			Benralizumab 2mg	16	Mean (SD) 0.21 (0.46)		0.57 (NR)	0.271
			Benralizumab 20mg	11	Mean (SD) 0.82 (0.91)		-0.70 (NR)	0.265
			Benralizumab 100mg	124	Mean (SD) 0.42 (0.65)		0.16 (-0.15-0.39)[9]	0.479
20	Jackson et al. 2020	Serum IgE concentration (kU/L) <62.0	Placebo	75	1.40 (1.00-1.96)	-	-	-
			Benralizumab Q8W	73	0.71 (0.46-1.09)	-0.69 (-1.21 to -0.17)	0.51 (0.31-0.84)	0.0079
		Serum IgE concentration (kU/L) ≥62.0 to <176.2	Placebo	112	1.66 (1.26-2.18)	-	-	-
			Benralizumab Q8W	109	0.79 (0.56-1.10)	-0.87 (-1.38 to -0.37)	0.47 (0.31-0.72)	0.0004
		Serum IgE concentration (kU/L) ≥176.2 to <453.4	Placebo	125	1.37 (1.07-1.76)	-	-	-
			Benralizumab Q8W	106	0.71 (0.52-0.97)	-0.67 (-1.06 to -0.27)	0.52 (0.35-0.76)	0.0008
		Serum IgE concentration (kU/L) >453.4	Placebo	129	1.22 (0.92-1.62)	-	-	-
			Benralizumab Q8W	128	0.68 (0.48-0.96)	-0.54 (-0.93 to -0.15)	0.56 (0.37-0.84)	0.0057
21	Chipps et al. 2020	FAO+	Placebo	308	1.32 (1.13-1.55)	-	-	-
			Benralizumab Q8W	313	0.74 (0.61-0.88)	-0.59 (-0.83 to -0.34)	0.56 (0.44-0.71)	<0.0001
		FAO-	Placebo	193	0.86 (0.69-1.08)	-	-	-
			Benralizumab Q8W	176	0.50 (0.38-0.66)	-0.36 (-0.60 to -0.12)	0.58 (0.41-0.83)	0.003
25		Winter	Benralizumab Q4W	505	0.91 (0.75-1.11)[10]		0.63 (0.49-0.81)[11]	<0.001

[10] Marginal Annual Exacerbation Rate (95%CI)
[11] Rate Ratio (95%CI)

List	Trial	Treatment arm		Subjects	Crude annual rate (95% CI)	Absolute difference estimate	Relative reduction vs. placebo	p value vs. placebo
	DuBuske et al. 2018		Benralizumab Q8W	495	0.87 (0.70-1.07)[10]		0.60 (0.46-0.78)[11]	<0.001
			Placebo	513	1.45 (1.23-1.70)[10]	-	-	-
		Spring	Benralizumab Q4W	505	0.66 (0.53-0.84) [10]		0.60 (0.44-0.81) [11]	<0.001
			Benralizumab Q8W	490	0.55 (0.42-0.72) [10]		0.50 (0.36-0.69) [11]	<0.001
			Placebo	504	1.11 (0.92-1.34) [10]	-	-	-
		Summer	Benralizumab Q4W	508	0.56 (0.43-0.73) [10]		0.54 (0.39-0.76) [11]	<0.001
			Benralizumab Q8W	487	0.57 (0.43-0.75) [10]		0.55 (0.39-0.78) [11]	<0.001
			Placebo	500	1.02 (0.83-1.27) [10]	-	-	-
		Fall	Benralizumab Q4W	506	0.88 (0.71-1.08) [10]		0.57 (0.44-0.74) [11]	<0.001
			Benralizumab Q8W	493	0.84 (0.67-1.04) [10]		0.54 (0.42-0.71) [11]	<0.001
			Placebo	510	1.54 (1.30-1.81) [10]	-	-	-
26	Chipps et al. 2018	Met atopy and IgE 30-700 kU/L criteria	Placebo	179	1.25 (1.01-1.54)	-	-	-
			Benralizumab 30mg Q4W	153	0.62 (0.47-0.81)	-0.63 (-0.93 to 0.32)	0.50 (0.36 to 0.69)	<0.0001
			Benralizumab 30mg Q8W	185	0.67 (0.52-0.86)	-0.58 (-0.89 to -0.27)	0.54 (0.39 to 0.74)	0.0002
		Did not meet atopy and IgE 30-700 kU/L criteria	Placebo	336	1.07 (0.91-1.26)			
			Benralizumab 30mg Q4W	363	0.69 (0.58-0.82)	-0.38 (-0.59 to -0.17)	0.64 (0.51 to 0.81)	0.0002
			Benralizumab 30mg Q8W	321	0.65 (0.54-0.78)	-0.42 (-0.63 to -0.21)	0.61 (0.47 to 0.78)	<0.0001
		IgE high (≥150 kU/L)	Placebo	304	1.09 (0.92-1.29)			
			Benralizumab 30mg Q4W	304	0.69 (0.58-0.84)	-0.39 (-0.62 to 0.17)	0.64 (0.50 to 0.82)	0.0004

List	Trial	Treatment arm		Subjects	Crude annual rate (95% CI)	Absolute difference estimate	Relative reduction vs. placebo	p value vs. placebo
			Benralizumab 30mg Q8W	297	0.63 (0.52-0.77)	-0.46 (-0.68 to -0.24)	0.58 (0.45 to 0.75)	<0.0001
		IgE low (<150 kU/L)	Placebo	206	1.21 (0.99-1.47)			
			Benralizumab 30mg Q4W	207	0.65 (0.52-0.82)	-0.56 (-0.84 to -0.27)	0.54 (0.40 to 0.73)	<0.0001
			Benralizumab 30mg Q8W	199	0.68 (0.54-0.87)	-0.52 (-0.82 to -0.23)	0.57 (0.41 to 0.78)	0.0002
		With atopy	Placebo	316	1.10 (0.94-1.30)			
			Benralizumab 30mg Q4W	307	0.71 (0.59-0.85)	-0.40 (-0.62 to -0.17)	0.64 (0.50 to 0.82)	0.0004
			Benralizumab 30mg Q8W	318	0.66 (0.55-0.80)	-0.44 (-0.66 to -0.22)	0.60 (0.47 to 0.77)	<0.0001
		Without atopy	Placebo	193	1.15 (0.95-1.41)			
			Benralizumab 30mg Q4W	201	0.60 (0.48-0.76)	-0.55 (-0.82 to -0.28)	0.52 (0.39 to 0.71)	<0.0001
			Benralizumab 30mg Q8W	181	0.62 (0.49-0.79)	-0.53 (-0.81 to -0.26)	0.54 (0.39 to 0.74)	0.0002
28	Ohta et al. 2018	[CALIMA Japan] High-dosage ICS plus LABA with baseline blood eosinophils ≥ 300 cells per uL	Placebo	16	2.45 (1.23-4.89)	-	-	-
			Benralizumab 30mg Q4W	15	0.83 (0.36-1.89)		0.34 (0.11 to 0.99)	
			Benralizumab 30mg Q8W	15	0.42 (0.15-1.18)		0.17 (0.05 to 0.60)	
29	Goldman et al. 2017	[SIROCCO] High-dosage ICS plus LABA with baseline blood	Placebo	306	1.50 (1.27-1.76)	-	-	-
			Benralizumab 30mg Q8W	325	0.87 (0.72-1.05)	-0.63 (-0.91 to -0.35)	0.58 (0.46 to 0.74)	<0.001

List	Trial	Treatment arm		Subjects	Crude annual rate (95% CI)	Absolute difference estimate	Relative reduction vs. placebo	p value vs. placebo
		eosinophils ≥ 150 cells per uL						
		[CALIMA] High-dosage ICS plus LABA with baseline blood eosinophils ≥ 150 cells per uL	Placebo	315	1.10 (0.94-1.28)	-	-	-
			Benralizumab 30mg Q8W	300	0.70 (0.58-0.85)	-0.40 (-0.61 to -0.19)	0.64 (0.50 to 0.81)	<0.001
		[SIROCCO] High-dosage ICS plus LABA with baseline blood eosinophils < 150 cells per uL	Placebo	74	1.34 (1.00-1.79)	-	-	-
			Benralizumab 30mg Q8W	48	1.01 (0.66-1.55)	-0.33 (-0.91 to 0.25)	0.76 (0.45 to 1.27)	0.287
		[CALIMA] High-dosage ICS plus LABA with baseline blood eosinophils < 150 cells per uL	Placebo	40	1.55 (1.06-2.28)	-	-	-
			Benralizumab 30mg Q8W	48	1.01 (0.69-1.48)	-0.54 (-1.23 to 0.14)	0.65 (0.39 to 1.09)	0.105

Table S7. Summary of FEV_1

List	Trial		Treatment arms	N	FEV1 before bronchodilation LS mean change	LS mean change (number of patients[12])	Difference vs. placebo	Difference vs. placebo (95% CI)	P-value
1	Menzies-Gow et al. 2021		Placebo	531	Mean (SD) 0.23 (0.02)	531	-	-	-
			Tezepelumab	528	Mean (SD) 0.09 (0.02)	528	0.13	0.08 to 0.18	P<0.001
4	Busse et al. 2018	≥ 300 cells per uL (week 48)	Q4W/Q4W group	347	-0.011 (0.323; -0.047-0.025)	308			
			Placebo/Q4W group	172	0.129 (0.449; 0.057-0.200)	150			
			Total	519	0.035 (0.374; 0.001-0.069)	458			

12 Number of patients with end of treatment data

List	Trial	Treatment arms		N	FEV1 before bronchodilation LS mean change	LS mean change (number of patients[12])	Difference vs. placebo	Difference vs. placebo (95% CI)	P-value
			Q8W/Q8W group	339	0.053 (0.345; 0.015-0.092)	310			
			Placebo/Q8W group	188	0.125 (0.402; 0.065-0.186)	169			
			Total	527	0.079 (0.367; 0.046-0.112)	469			
		< 300 cells per uL (week 48)	Q4W/Q4W group	171	0.016 (0.332; -0.037-0.069)	150			
			Placebo/Q4W group	93	0.017 (0.514; -0.094-0.127)	83			
			Total	264	0.016 (0.405; -0.036-0.068)	233			
			Q8W/Q8W group	173	0.001 (0.304; -0.046-0.048)	162			
			Placebo/Q8W group	93	0.073 (0.407; -0.016-0.162)	80			
			Total	266	0.025 (0.342; -0.018-0.068)	242			
		≥ 300 cells per uL (week 56[13])	Q4W/Q4W group	347	-0.006 (0.295; -0.039-0.028)	297			
			Placebo/Q4W group	172	0.131 (0.422; 0.061-0.200)	142			
			Total	519	0.038 (0.346; 0.006-0.071)	439			
			Q8W/Q8W group	339	0.019 (0.317; -0.018-0.055)	291			
			Placebo/Q8W group	188	0.081 (0.419; 0.014-0.148)	151			
			Total	527	0.040 (0.356; 0.007-0.073)	442			
		< 300 cells per uL (week 56[13])	Q4W/Q4W group	171	-0.021 (0.376; -0.081-0.040)	147			
			Placebo/Q4W group	93	-0.011 (0.280; -0.073-0.051)	79			

[13] Week 56 data are for adults only

List	Trial	Treatment arms		N	FEV1 before bronchodilation LS mean change	LS mean change (number of patients[12])	Difference vs. placebo	Difference vs. placebo (95% CI)	P-value
			Total	264	-0.017 (0.345; -0.062-0.028)	226			
			Q8W/Q8W group	173	-0.015 (0.293; -0.062-0.032)	14			
			Placebo/Q8W group	93	0.030 (0.350; -0.053-0.112)	69			
			Total	266	-0.001 (0.312; -0.042-0.040)	218			
5	Corren et al. 2017	Total	Placebo	131	-0.06	131	-		
			Low-dose Tezepelumab	130	0.07	130	0.12	0.02 to 0.22	0.015
			Medium-Dose Tezepelumab	121	0.08	121	0.13	0.03 to 0.23	0.009
			High-dose Tezepelumab	116	0.1	116	0.15	0.05 to 0.25	0.002
		≥ 250 Eosinophils per uL	Placebo	76	-0.05	76	-	-	-
			Tezepelumab 70mg q4w	77	0.12	77	0.16	0.03 to 0.29	0.014
			Tezepelumab 210mg q4w	66	0.13	66	0.17	0.04 to 0.3	0.013
			Tezepelumab 280mg q2w	63	0.16	63	0.21	0.07 to 0.34	0.003
		< 250 Eosinophils per uL	Placebo	55	-0.07	55	-	-	-
			Tezepelumab 70mg q4w	53	-0.03	53	0.04	-0.11 to 0.19	0.580
			Tezepelumab 210mg q4w	55	0.01	55	0.08	-0.07 to 0.23	0.289
			Tezepelumab 280mg q2w	53	0.01	53	0.08	-0.07 to 0.23	0.275
7	Bleecker et al. 2016	High-dosage ICS plus LABA with baseline blood eosinophils ≥	Placebo	261	0.239[14]	233	-	-	-
			Benralizumab 30mg Q4W	271	0.345[14]	236	0.106	0.016 to 0.196	0.0215
			Benralizumab 30mg Q8W	264	0.398[14]	235	0.159	0.068 to 0.249	0.0006

[14] Estimates calculated using a mixed-effects model for repeated measures analysis, with adjustment for treatment, baseline value, region, oral corticosteroid use at time of randomization, visit, and visit*treatment

List	Trial	Treatment arms		N	FEV1 before bronchodilation LS mean change	LS mean change (number of patients[12])	Difference vs. placebo	Difference vs. placebo (95% CI)	P-value
		300 cells per uL							
		High-dosage ICS plus LABA with baseline blood eosinophils < 300 cells per uL	Placebo	138	0.145[14]	125	-	-	-
			Benralizumab 30mg Q4W	120	0.120[14]	105	-0.025	-0.134 to 0.083	0.6438
			Benralizumab 30mg Q8W	129	0.248[14]	119	0.102	0.003 to 0.208	0.568
8	FitzGerald et al. 2016	High-dosage ICS plus LABA with baseline blood eosinophils ≥ 300 cells per uL	Placebo	244	0.215[14]	221	-	-	-
			Benralizumab 30mg Q4W	238	0.340[14]	216	0.125	0.037 to 0.213	0.0054
			Benralizumab 30mg Q8W	238	0.330[14]	211	0.116	0.028 to 0.204	0.0102
		High-dosage ICS plus LABA with baseline blood eosinophils < 300 cells per uL	Placebo	116	0.156[14]	99	-	-	-
			Benralizumab 30mg Q4W	114	0.219[14]	101	0.064	-0.049 to 0.176	0.2676
			Benralizumab 30mg Q8W	121	0.140[14]	98	-0.015	-0.127 to 0.096	0.7863
10	Castro et al. 2014	Eosinophilic	Placebo	51	Mean (SD) 0.04 (0.46)	51	-	-	-
			Benralizumab 2mg	51	Mean (SD) 0.16 (0.47)	51	0.13	0.02 to 0.24[9]	0.14
			Benralizumab 20mg	58	Mean (SD) 0.19 (0.52)	58	0.17	0.05 to 0.29[9]	0.069
			Benralizumab 100mg	59	Mean (SD) 0.17 (0.39)	59	0.15	0.05 to 0.25[9]	0.063
		Non-eosinophilic	Placebo	99	Mean (SD) -0.01 (0.30)	99	-	-	-
			Benralizumab 100mg	101	Mean (SD) 0.06 (0.33)	101	0.06	0.01 to 0.12[9]	0.155
		Baseline blood eosinophil count ≥300 cells per uL	Placebo	53	Mean (SD) -0.01	53	-	-	-
			Benralizumab 2mg	41	Mean (SD) 0.16	41	0.17	0.05 to 0.29[9]	0.079
			Benralizumab 20mg	48	Mean (SD) 0.2	48	0.23	0.11 to 0.36[9]	0.019
			Benralizumab 100mg	68	Mean (SD) 0.19	68	0.2	0.10 to 0.29[9]	0.01

List	Trial	Treatment arms		N	FEV1 before bronchodilation LS mean change	LS mean change (number of patients[12])	Difference vs. placebo	Difference vs. placebo (95% CI)	P-value
		Baseline blood eosinophil count <300 cells per uL	Placebo	97	Mean (SD) 0.02	97	-	-	-
			Benralizumab 2mg	10	Mean (SD) 0.17	10	-	-	-
			Benralizumab 20mg	10	Mean (SD) 0.11	10	-	-	-
			Benralizumab 100mg	91	Mean (SD) 0.04	91	0.02	-0.04 to 0.08[9]	0.637
21	Chipps et al. 2020	FAO+[15]	Placebo	304	0.184	277	-	-	-
			Benralizumab q8w	312	0.343	284	0.159	0.082 to 0.236	<0.0001
		FAO-	Placebo	190	0.293	168	-	-	-
			Benralizumab q8w	175	0.396	148	0.103	-0.008 to 0.215	0.0699
26	Chipps et al. 2018	Met atopy and IgE 30-700 kU/L criteria	Placebo	178	0.238	155	-	-	-
			Benralizumab 30mg Q4W	149	0.367	135	0.129	0.017 to 0.241	0.0244
			Benralizumab 30mg Q8W	184	0.363	161	0.125	0.018 to 0.232	0.0218
		Did not meet atopy and IgE 30-700 kU/L criteria	Placebo	327	0.217	299	-	-	-
			Benralizumab 30mg Q4W	360	0.330	317	0.114	0.040 to 0.187	0.0024
			Benralizumab 30mg Q8W	318	0.369	285	0.152	0.076 to 0.228	<0.0001
		IgE high (≥150 kU/L)	Placebo	301	0.247	268	-	-	-
			Benralizumab 30mg Q4W	299	0.367	267	0.120	0.038 to 0.202	0.0042
			Benralizumab 30mg Q8W	296	0.370	264	0.123	0.041 to 0.205	0.0034
		IgE low (<150 kU/L)	Placebo	200	0.200	182	-	-	-
			Benralizumab 30mg Q4W	205	0.297	181	0.098	0.004 to 0.191	0.0405
			Benralizumab 30mg Q8W	197	0.338	174	0.138	0.044 to 0.233	0.0041
		With atopy	Placebo	314	0.236	276	-	-	-
			Benralizumab 30mg Q4W	303	0.339	269	0.103	0.022 to 0.184	0.0124

[15] FAO+ and FAO- are defined as <70% or ≥70% of a ratio (*100) of postbronchodilator FEV_1 to FVC, respectively, at baseline, estimates calculated via a repeated measures model, with adjustment for study code, treatment, baseline value, region, OCS use at time of randomization, visit, and vist*treatment

List	Trial	Treatment arms		N	FEV1 before bronchodilation LS mean change	LS mean change (number of patients[12])	Difference vs. placebo	Difference vs. placebo (95% CI)	P-value
			Benralizumab 30mg Q8W	316	0.349	279	0.114	0.033 to 0.194	0.0056
		Without atopy	Placebo	186	0.201	173	-	-	-
			Benralizumab 30mg Q4W	198	0.348	176	0.148	0.053 to 0.242	0.0021
			Benralizumab 30mg Q8W	180	0.382	162	0.181	0.185 to 0.278	0.0002
28	Ohta et al. 2018	[CALIMA Japan] High-dosage ICS plus LABA with baseline blood eosinophils ≥ 300 cells per uL	Placebo	16			-	-	-
			Benralizumab 30mg Q4W	15		15	0.334	0.020 to 0.647	
			Benralizumab 30mg Q8W	15			0.198	-0.118 to 0.514	
29	Goldman et al. 2017	[SIROCCO] High-dosage ICS plus LABA with baseline blood eosinophils ≥ 150 cells per uL	Placebo	300	0.16	267	-	-	-
			Benralizumab 30mg Q8W	323	0.323	290	0.163	0.087 to 0.239	<0.001
		[CALIMA] High-dosage ICS plus LABA with baseline blood eosinophils ≥ 150 cells per uL	Placebo	308	0.169	282	-	-	-
			Benralizumab 30mg Q8W	298	0.286	258	0.116	0.041 to 0.191	0.002
		[SIROCCO] High-dosage ICS plus	Placebo	72	0.125	64	-	-	-
			Benralizumab 30mg Q8W	46	0.265	41	0.140	-0.045 to 0.325	0.136

List	Trial	Treatment arms		N	FEV1 before bronchodilation LS mean change	LS mean change (number of patients[12])	Difference vs. placebo	Difference vs. placebo (95% CI)	P-value
		LABA with baseline blood eosinophils < 150 cells per uL							
		[CALIMA] High-dosage ICS plus LABA with baseline blood eosinophils < 150 cells per uL	Placebo	37	0.164	33	-	-	-
			Benralizumab 30mg Q8W	46	0.033	40	0.131	-0.306 to 0.045	0.142

Table S8. Summary of Asthma symptoms score

List	Trial	Treatment arms		N	Total Asthma symptom score LS mean change	LS mean change (number of patients)	Difference vs. placebo	Difference vs. placebo (95% CI)	P-value
5	Ferguson et al. 2017		Placebo	105	-0.45	94	-	-	-
			Benralizuamb	104	-0.57	90	-0.13	-0.35 to 0.10	0.27
7	Bleecker et al. 2016	High-dosage ICS plus LABA with baseline blood eosinophils ≥ 300 cells per uL	Placebo	267	-1.04[14]	180	-	-	-
			Benralizumab 30mg Q4W	273	-1.12[14]	197	-0.08	-0.27 to 0.12	0.442
			Benralizumab 30mg Q8W	263	-1.3[14]	178	-0.25	-0.45 to -0.06	0.0118
		High-dosage ICS plus LABA with	Placebo	139	-0.77[14]	99	-	-	-
			Benralizumab 30mg Q4W	123	-0.97[14]	93	-0.20	-0.48 to 0.08	0.1688

List	Trial	Treatment arms		N	Total Asthma symptom score LS mean change	LS mean change (number of patients)	Difference vs. placebo	Difference vs. placebo (95% CI)	P-value
		baseline blood eosinophils < 300 cells per uL	Benralizumab 30mg Q8W	127	-1.06[14]	91	-0.29	-0.57 to -0.01	0.0431
8	FitzGerald et al. 2016	High-dosage ICS plus LABA with baseline blood eosinophils ≥ 300 cells per uL	Placebo	247	-1.16[16]	187	-	-	-
			Benralizumab 30mg Q4W	241	-1.28[16]	184	-0.12	-0.32 to 0.07	0.2241
			Benralizumab 30mg Q8W	237	-1.4[16]	185	-0.23	-0.43 to -0.04	0.0186
		High-dosage ICS plus LABA with baseline blood eosinophils < 300 cells per uL	Placebo	122	-0.95[16]	89	-	-	-
			Benralizumab 30mg Q4W	115	-1.11[16]	88	-0.16	-0.44 to 0.13	0.2868
			Benralizumab 30mg Q8W	124	-0.95[16]	85	-0.01	-0.28 to 0.29	0.9663
21	Chipps et al. 2020	FAO+	Placebo	308	-1.05	230	-	-	-
			Benralizumab q8w	311	-1.34	250	-0.13	-0.45 to -0.14	0.0002
		FAO-	Placebo	192	-1.26	129	-	-	-
			Benralizumab q8w	172	-1.33	102	-0.08	-0.30 to 0.14	0.4941
28	Ohta et al. 2018	[CALIMA Japan] High-dosage ICS plus LABA with baseline	Placebo				-	-	-
			Benralizumab 30mg Q4W	15			0.17	-0.82 to 0.48	
			Benralizumab 30mg Q8W	15			0.24	-0.87 to 0.40	

16 Key secondary endpoint; composite of daytime and night-time symptoms scored 0-6 overall (a decrese in score indicates improvement)

List	Trial	Treatment arms		N	Total Asthma symptom score LS mean change	LS mean change (number of patients)	Difference vs. placebo	Difference vs. placebo (95% CI)	P-value
		blood eosinophils ≥ 300 cells per uL							
29	Goldman et al. 2017	[SIROCCO] High-dosage ICS plus LABA with baseline blood eosinophils ≥ 150 cells per uL	Placebo	306	-0.95	213	-	-	-
			Benralizumab 30mg Q8W	320	-1.18	221	-0.23	-0.41 to -0.06	0.009
		[CALIMA] High-dosage ICS plus LABA with baseline blood eosinophils ≥ 150 cells per uL	Placebo	314	-1.08	242	-	-	-
			Benralizumab 30mg Q8W	298	-1.24	227	-0.16	-0.33 to 0.01	0.073
		[SIROCCO] High-dosage ICS plus LABA with baseline blood eosinophils < 150 cells per uL	Placebo	73	-0.8	50	-	-	-
			Benralizumab 30mg Q8W	47	-1.2	34	-0.40	-0.78 to -0.03	0.035
		[CALIMA] High-dosage ICS plus LABA with	Placebo	40	-0.84	29	-	-	-
			Benralizumab 30mg Q8W	48	-0.79	37	0.04	-0.37 to 0.45	0.842

List	Trial	Treatment arms		N	Total Asthma symptom score LS mean change	LS mean change (number of patients)	Difference vs. placebo	Difference vs. placebo (95% CI)	P-value
		baseline blood eosinophils < 150 cells per uL							

Table S9. Summary of ACQ-6 score, and AQLQ(S)+12 score

List	Trial	Treatment arm		ACQ-6 score N	ACQ-6 score Least-square mean change from baseline	ACQ-6 score Differenc e vs. placebo	ACQ-6 score P-value	AQLQ (S) +12 score N	AQLQ (S) +12 score Least-square mean change from baseline	AQLQ (S) +12 score Difference vs. placebo	AQLQ (S) +12 score P-value
1	Menzies-Gow et al.		Placebo	528	Mean (SD) -1.55(0.55)	-	-	254	Mean (SD) 3.9(1.0)	-	-
			Tezepelumab	531	Mean (SD) -1.22(0.05)	-0.33 (-0.46 to 0.20)	<0.001	261	Mean (SD) 3.9(1.0)	0.34 (0.20 to 0.47)	<0.001
4	Busse et al. 2018	≥ 300 cells per uL (week 48)	Q4W/Q4W group	320	0.01 (0.89; -0.09-0.10)			317	0.00 (0.90; -0.10-0.10)		
			Placebo/Q4W group	158	-0.27 (0.94; -0.42-0.13)			157	0.19 (0.95; 0.04-0.34)		
			Total	478	-0.09 (0.92; -0.17-0.00)			474	0.06 (0.92; -0.02-0.15)		
			Q8W/Q8W group	316	0.00 (0.81; -0.09-0.09)			316	0.02 (0.89; -0.08-0.12)		
			Placebo/Q8W group	173	-0.23 (1.07; -0.39-0.07)			171	0.26 (1.02; 0.10-0.41)		
			Total	489	-0.08 (0.92; -0.16-0.00)			487	0.10 (0.95; 0.02-0.19)		
		< 300 cells per uL (week 48)	Q4W/Q4W group	157	-0.19 (0.91; -0.33-0.05)			155	0.16 (0.84; 0.02-0.29)		
			Placebo/Q4W group	83	-0.04 (0.87; -0.23-0.14)			83	0.04 (1.00; -0.18-0.25)		

List	Trial	Treatment arm		ACQ-6 score N	ACQ-6 score Least-square mean change from baseline	ACQ-6 score Difference vs. placebo	ACQ-6 score P-value	AQLQ (S) +12 score N	AQLQ (S) +12 score Least-square mean change from baseline	AQLQ (S) +12 score Difference vs. placebo	AQLQ (S) +12 score P-value
			Total	240	-0.14 (0.90; -0.25-0.03)			238	0.12 (0.90; 0.00-0.23)		
			Q8W/Q8W group	164	-0.07 (0.79; -0.19-0.05)			164	0.14 (0.85; 0.01-0.27)		
			Placebo/Q8W group	85	-0.29 (0.98; -0.50-0.08)			85	0.08 (0.96; -0.12-0.29)		
			Total	249	-0.14 (0.86; -0.25-0.04)			249	0.12 (0.89; 0.01-0.23)		
		≥ 300 cells per uL (week 56)	Q4W/Q4W group	299	-0.04 (0.83; -0.14-0.05)			297	0.02 (0.80; -0.07-0.11)		
			Placebo/Q4W group	146	-0.20 (1.04; -0.37-0.03)			146	0.21 (0.98; 0.05-0.37)		
			Total	445	-0.09 (0.91; -0.18--0.01)			443	0.08 (0.87; 0.00-0.16)		
			Q8W/Q8W group	297	-0.06 (0.82; -0.15-0.03)			295	0.08 (0.91; -0.02-0.19)		
			Placebo/Q8W group	153	-0.25 (1.06; -0.41-0.08)			153	0.26 (1.00; 0.10-0.42)		
			Total	450	-0.12 (0.91; -0.21--0.04)			448	0.15 (0.94; 0.06-0.23)		
		< 300 cells per uL (week 56)	Q4W/Q4W group	149	-0.16 (0.90; -0.31--0.02)			148	0.11 (0.84; -0.02-0.25)		
			Placebo/Q4W group	79	-0.12 (0.80; -0.29-0.06)			79	0.03 (0.89; -0.16-0.23)		
			Total	228	-0.15 (0.86; -0.26--0.03)			227	0.09 (0.85; -0.03-0.20)		
			Q8W/Q8W group	150	-0.10 (0.83; -0.23 - 0.04)			149	0.15 (0.90; 0.00 - 0.29)		
			Placebo/Q8W group	67	-0.09 (1.06; -0.35-0.16)			67	0.02 (1.06; -0.23-0.28)		
			Total	217	-0.10 (0.90; -0.22-0.02)			216	0.11 (0.95; -0.02-0.24)		
5	Corren et al. 2017	Total	Placebo	53	-0.91	-		47	0.97	-	
			Low-dose Tezepelumab	52	-1.17	-0.26 (-0.52 to 0.01)	0.059	51	1.12	0.14 (-0.13 to 0.42)	0.309

List	Trial	Treatment arm		ACQ-6 score				AQLQ (S) +12 score			
				N	Least-square mean change from baseline	Difference vs. placebo	P-value	N	Least-square mean change from baseline	Difference vs. placebo	P-value
			Medium-Dose Tezepelumab	44	-1.2	-0.29 (-0.56 to -0.01)	0.039	41	1.17	0.2 (-0.09 to 0.48)	0.185
			High-dose Tezepelumab	49	-1.22	-0.31 (-0.58 to -0.04)	0.024	48	1.32	0.34 (0.06 to 0.63)	0.017
		≥ 250 Eosinophils per uL	Placebo	68	-0.89	-		62	0.90	-	
			Tezepelumab 70mg q4w	70	-1.08	-0.19 (-0.49 to 0.11)	0.207	69	1.05	0.15 (-0.19 to 0.48)	0.383
			Tezepelumab 210mg q4w	60	-1.37	-0.48 (-0.79 to -0.17)	0.002	54	1.31	0.41 (0.06 to 0.76)	0.022
			Tezepelumab 280mg q2w	55	-1.15	-0.27 (-0.58 to -0.05)	0.094	54	1.17	0.27 (-0.08 to 0.61)	0.134
		< 250 Eosinophils per uL	Placebo	44	-0.93	-		43	0.93	-	
			Tezepelumab 70mg q4w	46	-1.12	-0.19 (-0.53 to 0.14)	0.261	41	1.02	0.09 (-0.25 to 0.43)	0.610
			Tezepelumab 210mg q4w	50	-1.15	-0.22 (-0.56 to -0.11)	0.186	43	1.17	0.24 (-0.10 to 0.58)	0.173
			Tezepelumab 280mg q2w	47	-1.29	-0.36 (-0.70 to -0.02)	0.036	45	1.43	0.49 (0.15 to 0.83)	0.004
7	Bleecker et al. 2016	High-dosage ICS /LABA with baseline blood eosinophils ≥ 300 cells per uL	Placebo	267	-1.17	-	-	254	1.26	-	-
			Benralizumab 30mg q4w	274	-1.32	-0.15 (-0.34 to 0.04)	0.1107	261	1.44	0.18 (-0.02 to 0.37)	0.0811
			Benralizumab 30mg q8w	263	-1.46	-0.29 (-0.48 to -0.10)	0.0028	252	1.56	0.3 (0.10 to 0.50)	0.0036

List	Trial	Treatment arm		ACQ-6 score N	ACQ-6 score Least-square mean change from baseline	ACQ-6 score Difference vs. placebo	ACQ-6 score P-value	AQLQ (S) +12 score N	AQLQ (S) +12 score Least-square mean change from baseline	AQLQ (S) +12 score Difference vs. placebo	AQLQ (S) +12 score P-value
		High-dosage ICS /LABA with baseline blood eosinophils < 300 cells per uL	Placebo	138	-0.89	-	-	-	-	-	-
			Benralizumab 30mg q4w	124	-0.89	0 (-0.27 to 0.27)	0.9903	-	-	-	-
			Benralizumab 30mg q8w	130	-1.11	-0.22 (-0.48 to - 0.05)	0.1066	-	-	-	-
8	FitzGerald et al. 2016	High-dosage ICS /LABA with baseline blood eosinophils ≥ 300 cells per uL	Placebo	247	-1.19	-	-	240	1.31	-	-
			Benralizumab 30mg Q4W	241	-1.38	-0.19 (-0.38 to - 0.01)	0.0425	233	1.47	0.16 (-0.04 to 0.37)	0.1194
			Benralizumab 30mg Q8W	239	-1.44	-0.25 (-0.44 to - 0.07)	0.0082	230	1.56	0.24 (0.04 to 0.45)	0.1194
		High-dosage ICS/LABA with baseline blood eosinophils < 300 cells per uL	Placebo	122	-0.89	-	-	-	-	-	-
			Benralizumab 30mg Q4W	116	-1.14	-0.24 (-0.51 to 0.03)	0.0776	-	-	-	-
			Benralizumab 30mg Q8W	125	-1.00	-0.10 (-0.37 to - 0.16)	0.4488	-	-	-	-
10	Castro et al. 2014	Eosinophilic	Placebo	34	Mean (SD) - 0.89(1.20)	-	-	-	-	-	-
			Benralizumab 2mg	42	Mean (SD) - 1.1(1.12)	-0.36 (-0.65 to - 0.06)[9]	0.125	-	-	-	-
			Benralizumab 20mg	40	Mean (SD) - 1.25(1.22)	-0.45 (-0.77 to - 0.13)[9]	0.074	-	-	-	-

List	Trial	Treatment arm		ACQ-6 score				AQLQ (S) +12 score			
				N	Least-square mean change from baseline	Difference vs. placebo	P-value	N	Least-square mean change from baseline	Difference vs. placebo	P-value
			Benralizumab 100mg	39	Mean (SD) -1.12(1.29)	-0.47 (-0.79 to -0.16)[9]	0.057	-	-	-	-
		Non-eosinophilic	Placebo	64	Mean (SD) -0.82(1.11)	-	-	-	-	-	-
			Benralizumab 100mg	73	Mean (SD) -1.13(1.05)	-0.34 (-0.57 to -0.12)[9]	0.053	-	-	-	-
		Baseline blood eosinophil count ≥300 cells per uL	Placebo	38	Mean (SD) -0.76 (1.20)	-	-	37	Mean (SD) 0.98 (1.44)	-	-
			Benralizumab 2mg	35	Mean (SD) -1.00 (1.14)	-0.34 (-0.65 to -0.03)[9]	0.156	32	Mean (SD) 1.08 (1.15)	0.26 (-0.09 to 0.62)[9]	0.335
			Benralizumab 20mg	35	Mean (SD) -1.14 (1.19)	-0.44 (-0.75 to -0.12)[9]	0.079	34	Mean (SD) 1.41 (1.46)	0.44 (0.06 to 0.81)[9]	0.134
			Benralizumab 100mg	52	Mean (SD) -1.26 (1.28)	-0.53 (-0.81 to -0.26)[9]	0.015	47	Mean (SD) 1.12 (1.23)	0.15 (-0.18 to 0.49)[9]	0.552
		Baseline blood eosinophil count <300 cells per uL	Placebo	60	Mean (SD) -0.89 (1.10)	-	-	51	Mean (SD) 0.95 (1.26)	-	-
			Benralizumab 2mg	7	Mean (SD) -1.62 (0.91)	-	-	7	Mean (SD) 2.11 (1.17)	-	-
			Benralizumab 20mg	5	Mean (SD) -2.00 (1.30)	-	-	4	Mean (SD) 1.80 (1.22)	-	-
			Benralizumab 100mg	60	Mean (SD) -1.02 (0.98)	-0.22(-0.46 to -0.02)[9]	0.233	58	Mean (SD) 1.13 (1.30)	0.31(0 to 0.61)[9]	0.195
21	Chipps et al. 2020	FAO+	Placebo	308	-1.14	-	-	299	1.21	-	-
			Benralizumab q8w	311	-1.47	-0.33 (-0.49 to -0.17)	<0.0001	300	1.55	0.33 (0.15 to 0.51)	0.0003
		FAO-	Placebo	192	-1.23	-	-	181	1.39	-	-
			Benralizumab q8w	175	-1.41	-0.18 (-0.40 to 0.04)	0.1096	166	1.55	0.17 (-0.08 to 0.41)	0.1894

List	Trial	Treatment arm		ACQ-6 score N	ACQ-6 score Least-square mean change from baseline	ACQ-6 score Difference vs. placebo	ACQ-6 score P-value	AQLQ (S) +12 score N	AQLQ (S) +12 score Least-square mean change from baseline	AQLQ (S) +12 score Difference vs. placebo	AQLQ (S) +12 score P-value
26	Chipps et al. 2018	Met atopy and IgE 30-700 kU/L criteria	Placebo	179	-1.07	-	-	176	1.23	-	-
			Benralizumab 30mg Q4W	152	-1.40	-0.33 (-0.55 to -0.11)	0.0038	147	1.51	0.28 (0.04 to 0.52)	0.0207
			Benralizumab 30mg Q8W	185	-1.41	-0.34 (-0.55 to -0.13)	0.0017	176	1.5	0.27 (0.04 to 0.50)	0.0193
		Did not meet atopy and IgE 30-700 kU/L criteria	Placebo	335	-1.22	-	-	318	1.32	-	-
			Benralizumab 30mg Q4W	363	-1.34	-0.12 (-0.28 to 0.03)	0.1111	347	1.43	0.11 (-0.06 to 0.27)	0.2057
			Benralizumab 30mg Q8W	317	-1.47	-0.26 (-0.41 to -0.10)	0.0016	306	1.59	0.27 (0.10 to 0.44)	0.0022
29	Goldman et al. 2017	[SIROCCO] High-dosage ICS/LABA with baseline blood eosinophils ≥ 150 cells per uL	Placebo	305	-1.13	-	-	294	1.18	-	-
			Benralizumab 30mg Q8W	321	-1.27	-0.15 (-0.31 to 0.02)	0.084	307	1.37	0.19 (0.01 to 0.37)	0.0036
		[CALIMA] High-dosage ICS/LABA with baseline blood eosinophils ≥ 150 cells per uL	Placebo	314	-1.08	-	-	305	1.19	-	-
			Benralizumab 30mg Q8W	300	-1.3	-0.22 (-0.39 to -0.06)	0.0008	292	1.39	0.2 (0.02 to 0.38)	0.029
			Placebo	73	-0.62			70	0.77		

List	Trial	Treatment arm		ACQ-6 score				AQLQ (S) +12 score			
				N	Least-square mean change from baseline	Difference vs. placebo	P-value	N	Least-square mean change from baseline	Difference vs. placebo	P-value
		[SIROCCO] High-dosage ICS/LABA with baseline blood eosinophils < 150 cells per uL	Benralizumab 30mg Q8W	47	-1.32	-0.7 (-1.15 to -0.25)	0.0003	46	1.24	0.46 (-0.01 to 0.94)	0.056
		[CALIMA] High-dosage ICS/LABA with baseline blood eosinophils < 150 cells per uL	Placebo	40	-0.84	-	-	39	0.98	-	-
			Benralizumab 30mg Q8W	48	-0.91	-0.07 (-0.56 to 0.43)	0.783	46	0.97	-0.01 (-0.48 to 0.47)	0.972

Table S10. Summary of ED visits/hospitalization

List	Trial	Treatment arm		Subjects	Rate estimate (95% CI)	Absolute difference estimate	Rate ratio vs. placebo	p value vs. placebo
**ED Visits/ hospitalization**								
11	Chipps et al. 2020	FAO+	Placebo	308	0.13 (0.09-0.18)[17]			
			Benralizumab Q8W	313	0.07 (0.05-0.11)	-0.06 (-0.11 to -0.01)	0.55 (0.33-0.91)	0.0195
		FAO-	Placebo	193	0.10 (0.06-0.16)			

[17] Any exacerbations resulting in ED visits or hospitalizations in the previous year (for annual exacerbation rate associated with ED visits or hospitalizations.

			Benralizumab Q8W	176	0.07 (0.04-0.13)	-0.03 (-0.09 to 0.03)	0.70 (0.33-1.48)	0.3514
21	Goldman et al. 2017	[SIROCCO] High-dosage ICS plus LABA with baseline blood eosinophils ≥ 150 cells per uL	Placebo	306	0.03 (0.02-0.05)			
			Benralizumab 30mg Q8W	325	0.03 (0.02-0.08)	-0.10 (-0.19 to -0.02)	0.54 (0.32-0.90)	0.018
		[CALIMA] High-dosage ICS plus LABA with baseline blood eosinophils ≥ 150 cells per uL	Placebo	315	NC			
			Benralizumab 30mg Q8W	300	NC	NC	NC	NC
		[SIROCCO] High-dosage ICS plus LABA with baseline blood eosinophils < 150 cells per uL	Placebo	74	0.16 (0.09-0.30)			
			Benralizumab 30mg Q8W	48	0.31 (0.15-0.64)	0.15 (-0.10 to 0.40)	1.92 (0.72-5.14)	0.192
		[CALIMA] High-dosage ICS plus LABA with baseline blood eosinophils < 150 cells per uL	Placebo	40	NC			
			Benralizumab 30mg Q8W	48	NC	NC	NC	NC
**ED Visits**								
7	Corren et al. 2020	Placebo		138				
		Low-dose Tezepelumab		138			0.44 (0.14-1.41)	
		Medium-Dose Tezepelumab		137			0.16 (0.04-0.69)	
		High-dose Tezepelumab		137			0.63 (0.22-1.81)	
		Overall tezepelumab		412			0.40 (0.17-0.97)	
14	Busse et al. 2018	≥ 300 cells per uL	Q4W/Q4W group	347	0.03 (0.02-0.05)			
			Placebo/Q4W group	172	0.03 (0.02-0.08)			
			Total	519	0.03 (0.02-0.05)			
			Q8W/Q8W group	339	0.04 (0.03-0.07)			

			Placebo/Q8W group	188	0.06 (0.04-0.11)			
			Total	527	0.05 (0.03-0.07)			
		< 300 cells per uL	Q4W/Q4W group	171	0.03 (0.01-0.07)			
			Placebo/Q4W group	93	0.04 (0.02-0.11)			
			Total	264	0.03 (0.02-0.07)			
			Q8W/Q8W group	173	0.03 (0.01-0.07)			
			Placebo/Q8W group	93	0.04 (0.02-0.11)			
			Total	266	0.04 (0.02-0.07)			
**Hospitalization**								
7	Corren et al. 2020	Placebo		138				
		Low-dose Tezepelumab		138			0.27 (0.09-0.76)	
		Medium-Dose Tezepelumab		137			0.52 (0.19-1.41)	
		High-dose Tezepelumab		137			0.42 (0.15-1.20)	
		Overall tezepelumab		412			0.39 (0.18-0.85)	
14	Busse et al. 2018	≥ 300 cells per uL	Q4W/Q4W group	347	0.05 (0.03-0.07)			
			Placebo/Q4W group	172	0.04 (0.02-0.08)			
			Total	519	0.04 (0.03-0.07)			
			Q8W/Q8W group	339	0.03 (0.02-0.06)			
			Placebo/Q8W group	188	0.05 (0.03-0.10)			
			Total	527	0.04 (0.03-0.06)			
		< 300 cells per uL	Q4W/Q4W group	171	0.05 (0.03-0.10)			

			Placebo/Q4W group	93	0.09 (0.04-0.17)			
			Total	264	0.06 (0.04-0.10)			
			Q8W/Q8W group	173	0.05 (0.03-0.10)			
			Placebo/Q8W group	93	0.05 (0.02-0.13)			
			Total	266	0.05 (0.03-0.09)			

Table S11. Summary of Observational Studies

List	Trial	Treatment Arm	N	Age at first assessment		Age at onset of symptoms		Gender		BMI	
				mean, (SD)	p-value	mean, (SD)	p-value	Female	p-value	mean, (SD)	P-value
13	Jackson et al. 2021	No biologic therapy	687	49.1 (14.3)	0.332	23.6 (18.9)	0.321	M:259 F:428	0.964	30.8 (7.4)	0.747
		Biologic therapy	1524	49.7 (14.3)		24.5 (19.2)		M:573 F:951		30.9 (6.9)	
		Anti-IgE	329	47.6 (14.6)	<0.001	15.2 (16.0)	<0.001	M:109 F:220	0.053	31.3 (6.8)	0.183
		Anti-IL5	1120	50.7 (14.1)		27.5 (19.2)		M:437 F:683		30.7 (7.0)	
		T2-low	210	48.1 (15.1)	0.522	17.1 (16.6)	<0.001	M:69 F:141	0.076	32.1 (7.8)	<0.001
		T2-high	992	48.8 (14.3)		25.9 (19.1)		M:391 F:601		30.2 (6.7)	
14	Padilla-Galo et al. 2020	Total	42	53.6 (11)				M:9 F:33		28.6 (6)	
		Biologic treatment naiive patients	20	58 (20.75)	0.089	30 (24)	0.079	M:4 F:16	1	28 (6.75)	0.98
		Patients with prior biologic therapy	22	52 (14.75)		22.5 (14)		M:5 F:17		27.5 (8.5)	
		Non-atopic	28	56 (20)	0.153	26.5 (23)	0.913	M:5 F:23	0.451	28.5 (7.5)	0.131

		Atopic	14	49.5 (15)		24.5 (16)		M:4<br>F:10		25 (10)	

Reference

1. Menzies-Gow, A., et al., *Tezepelumab in Adults and Adolescents with Severe, Uncontrolled Asthma.* N Engl J Med, 2021. **384**(19): p. 1800-1809.
2. Emson, C., et al., *CASCADE: a phase 2, randomized, double-blind, placebo-controlled, parallel-group trial to evaluate the effect of tezepelumab on airway inflammation in patients with uncontrolled asthma.* Respir Res, 2020. **21**(1): p. 265.
3. Busse, W.W., et al., *Long-term safety and efficacy of benralizumab in patients with severe, uncontrolled asthma: 1-year results from the BORA phase 3 extension trial.* Lancet Respir Med, 2019. **7**(1): p. 46-59.
4. Corren, J., et al., *Tezepelumab in Adults with Uncontrolled Asthma.* N Engl J Med, 2017. **377**(10): p. 936-946.
5. Nair, P., et al., *Oral Glucocorticoid-Sparing Effect of Benralizumab in Severe Asthma.* N Engl J Med, 2017. **376**(25): p. 2448-2458.
6. Bleecker, E.R., et al., *Efficacy and safety of benralizumab for patients with severe asthma uncontrolled with high-dosage inhaled corticosteroids and long-acting beta2-agonists (SIROCCO): a randomised, multicentre, placebo-controlled phase 3 trial.* Lancet, 2016. **388**(10056): p. 2115-2127.
7. FitzGerald, J.M., et al., *Benralizumab, an anti-interleukin-5 receptor alpha monoclonal antibody, as add-on treatment for patients with severe, uncontrolled, eosinophilic asthma (CALIMA): a randomised, double-blind, placebo-controlled phase 3 trial.* Lancet, 2016. **388**(10056): p. 2128-2141.
8. Park, H.S., et al., *A Phase 2a Study of Benralizumab for Patients with Eosinophilic Asthma in South Korea and Japan.* Int Arch Allergy Immunol, 2016. **169**(3): p. 135-45.
9. Castro, M., et al., *Benralizumab, an anti-interleukin 5 receptor alpha monoclonal antibody, versus placebo for uncontrolled eosinophilic asthma: a phase 2b randomised dose-ranging study.* Lancet Respir Med, 2014. **2**(11): p. 879-890.
10. Nowak, R.M., et al., *A randomized trial of benralizumab, an antiinterleukin 5 receptor alpha monoclonal antibody, after acute asthma.* Am J Emerg Med, 2015. **33**(1): p. 14-20.
11. Harrison, T.W., et al., *Onset of effect and impact on health-related quality of life, exacerbation rate, lung function, and nasal polyposis symptoms for patients with severe eosinophilic asthma treated with benralizumab (ANDHI): a randomised, controlled, phase 3b trial.* Lancet Respir Med, 2021. **9**(3): p. 260-274.
12. Laviolette, M., et al., *Effects of benralizumab on airway eosinophils in asthmatic patients with sputum eosinophilia.* J Allergy Clin Immunol, 2013. **132**(5): p. 1086-1096 e5.
13. Jackson, D.J., et al., *Characterisation of patients with severe asthma in the UK Severe Asthma Registry in the biologic era.* Thorax, 2021. **76**(3): p. 220-227.
14. Padilla-Galo, A., et al., *Real-life experience with benralizumab during 6 months.* BMC Pulm Med, 2020. **20**(1): p. 184.
15. Pelaia, C., et al., *Real-Life effects of benralizumab on exacerbation number and lung hyperinflation in atopic patients with severe eosinophilic asthma.* Biomed Pharmacother, 2020. **129**: p. 110444.
16. Menzella, F., et al., *Biologics and Bronchial Thermoplasty for severe refractory asthma treatment: From eligibility criteria to real practice. A cross-sectional study.* Pulm Pharmacol Ther, 2020. **60**: p. 101874.
17. Corren, J., et al., *Tezepelumab improves patient-reported outcomes in patients with severe, uncontrolled asthma in*

PATHWAY. Ann Allergy Asthma Immunol, 2021. **126**(2): p. 187-193.
18. Corren, J., et al., *The effect of tezepelumab on hospitalizations and emergency department visits in patients with severe asthma.* Ann Allergy Asthma Immunol, 2020. **125**(2): p. 211-214.
19. Lugogo, N.L., et al., *Blood eosinophil count group shifts and kinetics in severe eosinophilic asthma.* Ann Allergy Asthma Immunol, 2020. **125**(2): p. 171-176.
20. Jackson, D.J., et al., *Ability of Serum IgE Concentration to Predict Exacerbation Risk and Benralizumab Efficacy for Patients with Severe Eosinophilic Asthma.* Adv Ther, 2020. **37**(2): p. 718-729.
21. Chipps, B.E., et al., *Benralizumab efficacy for patients with fixed airflow obstruction and severe, uncontrolled eosinophilic asthma.* Ann Allergy Asthma Immunol, 2020. **124**(1): p. 79-86.
22. Chia, Y.L., et al., *Relationship Between Benralizumab Exposure and Efficacy for Patients With Severe Eosinophilic Asthma.* Clin Pharmacol Ther, 2019. **106**(2): p. 383-390.
23. Chupp, G., et al., *Rapid onset of effect of benralizumab on morning peak expiratory flow in severe, uncontrolled asthma.* Ann Allergy Asthma Immunol, 2019. **122**(5): p. 478-485.
24. Bleecker, E.R., et al., *Baseline patient factors impact on the clinical efficacy of benralizumab for severe asthma.* Eur Respir J, 2018. **52**(4).
25. DuBuske, L., et al., *Seasonal variability of exacerbations of severe, uncontrolled eosinophilic asthma and clinical benefits of benralizumab.* Allergy Asthma Proc, 2018. **39**(5): p. 345-349.
26. Chipps, B.E., et al., *Benralizumab efficacy by atopy status and serum immunoglobulin E for patients with severe, uncontrolled asthma.* Ann Allergy Asthma Immunol, 2018. **120**(5): p. 504-511 e4.
27. Sehmi, R., et al., *Benralizumab attenuates airway eosinophilia in prednisone-dependent asthma.* J Allergy Clin Immunol, 2018. **141**(4): p. 1529-1532 e8.
28. Ohta, K., et al., *Efficacy and safety of benralizumab in Japanese patients with severe, uncontrolled eosinophilic asthma.* Allergol Int, 2018. **67**(2): p. 266-272.
29. Goldman, M., et al., *The association between blood eosinophil count and benralizumab efficacy for patients with severe, uncontrolled asthma: subanalyses of the Phase III SIROCCO and CALIMA studies.* Curr Med Res Opin, 2017. **33**(9): p. 1605-1613.
30. Pham, T.H., et al., *Reductions in eosinophil biomarkers by benralizumab in patients with asthma.* Respir Med, 2016. **111**: p. 21-9.
